# Primary Pulmonary NUT Carcinoma: A Case Illustration of Therapeutic Challenges and Review of Emerging Targeted Therapies

**DOI:** 10.1155/crom/4908133

**Published:** 2026-04-07

**Authors:** Jessica Y. Bae, Xiang Yu Gao, Josephine K. Dermawan, Jessica A. Hellyer

**Affiliations:** ^1^ Elson S. Floyd College of Medicine, Washington State University, Spokane, Washington, USA, wsu.edu; ^2^ Robert J. Tomsich Pathology and Laboratory Medicine Institute, Cleveland Clinic, Cleveland, Ohio, USA, clevelandclinic.org; ^3^ San Juan Cancer Center, Montrose Regional Health, Montrose, Colorado, USA

**Keywords:** BRD4-NUT fusion, case report, lung cancer, NUT carcinoma, NUTM1 protein, PD-1 inhibitor

## Abstract

**Background:**

Pulmonary NUT carcinoma is a rare but highly aggressive malignancy with poor prognosis. It typically affects younger patients with no smoking history. Given its rapid progression, it is crucial to consider it as a differential diagnosis in a poorly differentiated thoracic mass to ensure timely diagnosis and management.

**Case Presentation:**

We report a case of primary pulmonary NUT carcinoma diagnosed in a 59‐year‐old male with a never‐smoking history during routine screening for possible occupational asbestos exposure. The preliminary diagnosis of metastatic keratinizing squamous cell carcinoma was later reclassified as NUT carcinoma by immunohistochemistry. Next generation sequencing of ctDNA showed *CDKN2A* mutation. The therapeutic course was complicated by several hypersensitivity reactions to first line treatments. Due to rapidly deteriorating clinical status, the patient was no longer eligible for a BET inhibitor clinical trial and died approximately 5 months after diagnosis.

**Conclusion:**

Due to its rarity and poor therapeutic response, there is currently no established standard of treatment for pulmonary NUT carcinoma. Timely and accurate diagnosis remains challenging due to its nonspecific presentation and rarity, resulting in low clinical suspicion, especially in patients outside the typical demographic. Common first‐line treatments include platinum‐based regimens in combination with etoposide or paclitaxel. Several clinical trials of BET and histone deacetylase inhibitors are active, and clinicians are encouraged to enroll patients to maximize survival outcomes.

## 1. Introduction

Nuclear protein of the testis (NUT) carcinoma is a rare and aggressive malignancy of the human squamous epithelium. Nuclear protein of the testis carcinoma (NC) typically affects the midline structures, including the thoracic and mediastinal organs and the head and neck regions [1]. Diagnosis is typically made in young adulthood, often before the age of 40. A substantial number of affected patients report a never‐smoking history [2]. However, the presence of a smoking history does not rule out the diagnosis. As with many lung malignancies, the initial clinical presentation is often nonspecific, most commonly with persistent cough and pleuritic chest pain, with CT findings revealing a large primary mass [2]. Additionally, it has a poor prognosis with a median overall survival of 6.7 months for NC and 2.2 months for primary pulmonary NC [3].

Following the initial detection of lung mass, diagnostic testing includes immunohistochemistry (IHC) for NUT protein overexpression and molecular techniques such as fluorescence in situ hybridization (FISH) or next‐generation sequencing (NGS) for *NUTM1* gene rearrangements [3, 4]. In contrast to the predominantly younger age group reported in the literature, we present a case of primary pulmonary NC in a 59‐year‐old male.

## 2. Case Presentation

A 59‐year‐old male was referred for evaluation of a new mass in the left lower lobe detected on surveillance imaging. The patient had no history of smoking or pre‐existing lung disease. The patient′s medical history is notable for occupational asbestos exposure, for which he received yearly chest x‐ray screenings. Imaging from the year prior was normal, without any masses or lesions. Aside from a chronic, intermittent nonproductive cough persisting for the past 6–8 months, the patient was asymptomatic.

PET/CT findings confirmed a hypermetabolic left lower lobe mass measuring 9.1 cm (Figures 1 and 2), ipsilateral pleural effusion, pleural rind, a hypermetabolic node at the right posterior cervical angle, and a mixed lytic‐sclerotic lesion of the left fifth rib with a pathologic fracture. Fine needle aspiration yielded a preliminary diagnosis of a metastatic keratinizing squamous cell carcinoma. A chest CT obtained 6 weeks later showed growth of the left lower lobe mass to 12.1 cm (previously 9.1 cm) and the lytic lesion of the left fifth rib, now measurable at 4.0 cm. The left fifth rib lesion was biopsied. Histologic examination revealed involvement of lamellar bone trabeculae by nests of epithelial cells with high nuclear‐to‐cytoplasmic ratios and prominent nucleoli, with foci of abrupt squamous keratinization (Figure 3). After IHC stains confirmed the diagnosis of metastatic NC of the lung, positive for p40 and NUT (speckled nuclear positivity) with weak CK AE1/AE3 and CAM 5.2 staining, the treatment plan was modified to carboplatin/etoposide followed by radiation for rib pain. A blood‐based circulating tumor DNA (ctDNA) NGS showed *CDKN2A* mutation.

Figure 1Initial chest CT of the patient (a: axial, b: sagittal) showed left lower lobe perihilar mass measuring 8.7 × 7.5 × 7.1 cm (red arrow).

**Figure:**
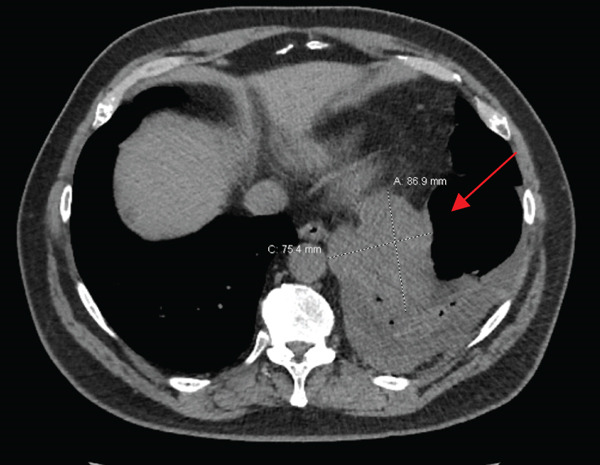
(a)

**Figure:**
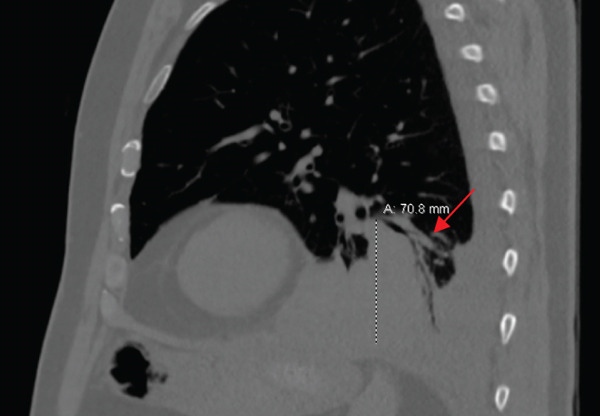
(b)

Figure 2Initial PET/CT taken 1 week after the CT (Figure 1 above) showed: (a) metabolic lesion in the posterior left fifth rib and (b) hypermetabolic cervical nodes, most prominently in the right posterior cervical area, concerning for metastatic disease process.

**Figure:**
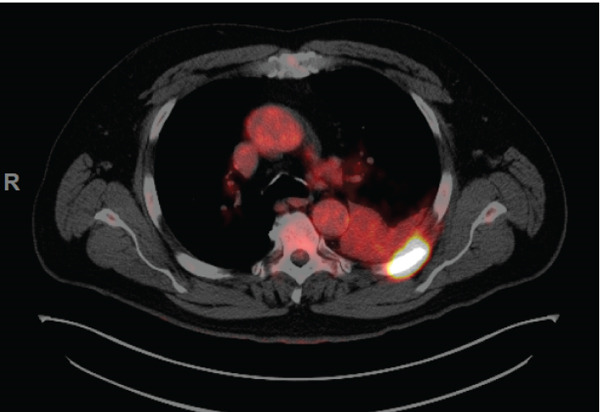
(a)

**Figure:**
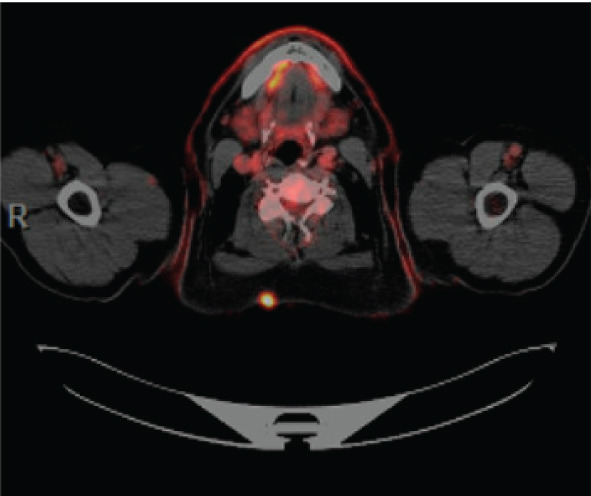
(b)

Figure 3Histologic examination of left fifth rib lesion biopsy. (a) Intermediate magnification shows tumor infiltration into lamellar bone trabeculae. (b, c) High‐power views reveal nests of poorly differentiated epithelial cells with high nuclear‐to‐cytoplasmic ratios and prominent nucleoli. Foci of squamous differentiation with abrupt keratinization are also present, consistent with common histologic features of NC. (d) Immunohistochemistry demonstrates diffuse speckled nuclear positivity for NUT, confirming the diagnosis of metastatic NC.

**Figure:**
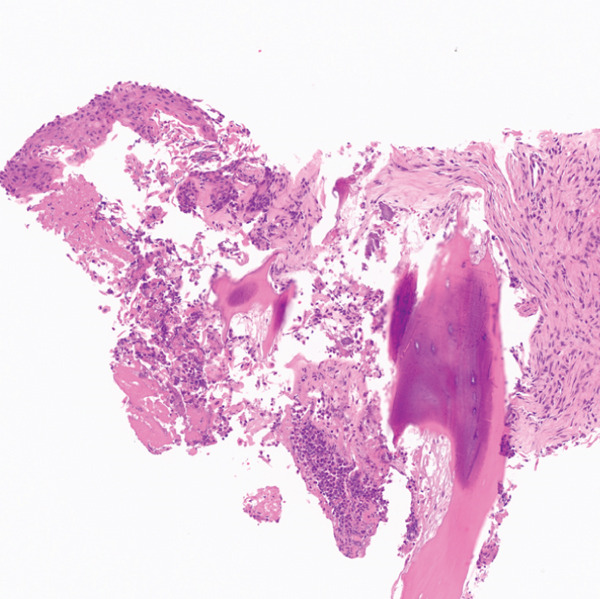
(a)

**Figure:**
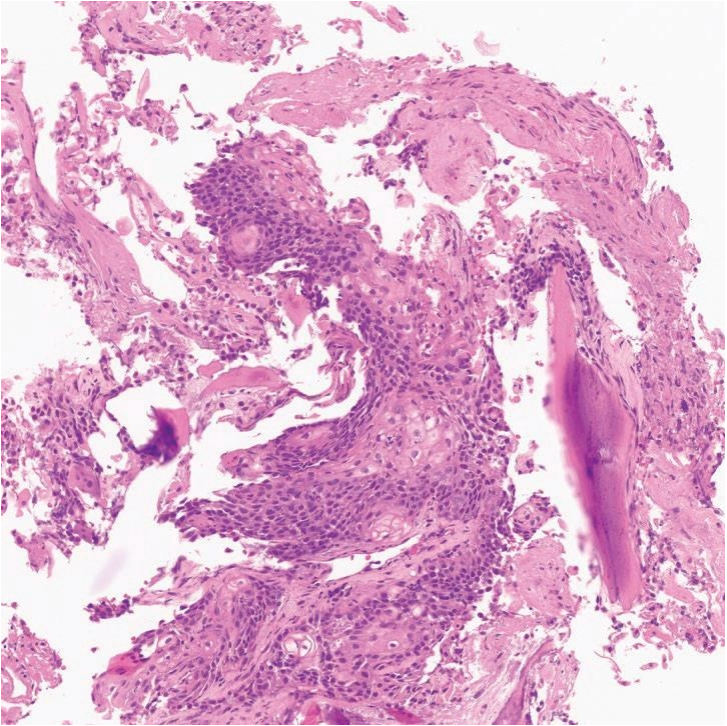
(b)

**Figure:**
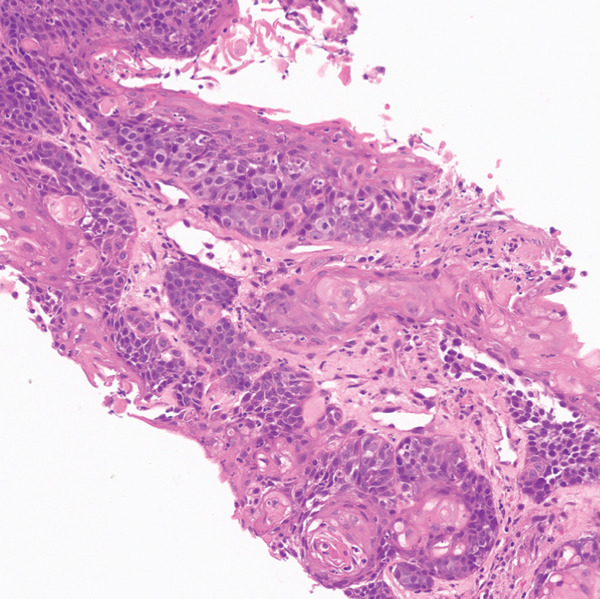
(c)

**Figure:**
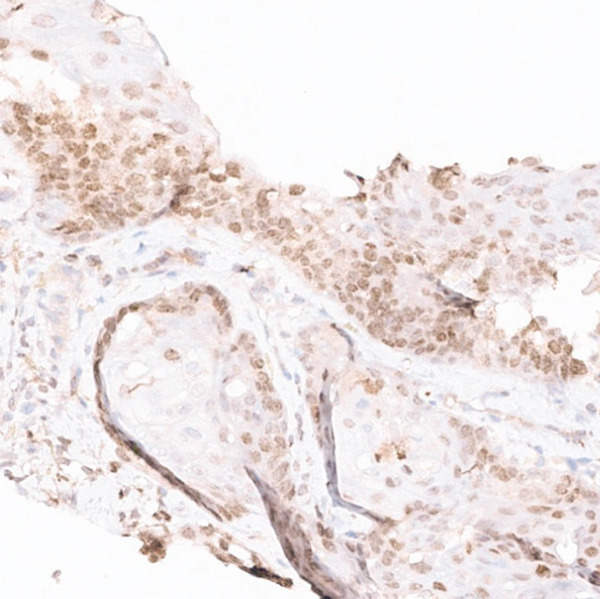
(d)

After the initial diagnosis of squamous cell carcinoma and prior to confirmatory testing for NC, the patient initiated the first cycle of carboplatin, paclitaxel, and pembrolizumab. Despite premedication with methylprednisolone, diphenhydramine, and famotidine, Cycle 1 therapy was complicated by a severe hypersensitivity reaction to paclitaxel, manifesting as hypoxia, cough, chest pressure, dyspnea, and flushing. The patient was then switched to carboplatin/etoposide but subsequently developed anaphylaxis to etoposide, leading to a therapy switch to carboplatin/gemcitabine with premedication including dexamethasone and montelukast. Additionally, the patient was evaluated for a clinical trial involving BET inhibitor (BETi) and CDK4/6 inhibitor (abemaciclib) combination therapy.

The disease course was further complicated by hospitalization for sepsis secondary to empyema. Despite four cycles of carboplatin with gemcitabine, the disease was poorly responsive to therapy with rapid progression of the primary tumor and new lesions. The last CT scan showed growth of the left lung mass to 13.8 cm and a left posteroinferior pleural mass measuring 4.1 cm (previously 3.2 cm when first detected 5 weeks prior). The patient′s clinical status continued to deteriorate since the hospitalization. Given the rapidly deteriorating performance status, the patient was unfortunately no longer a candidate for BETi clinical trial. Following discharge, the patient transitioned to palliative hospice support and died approximately 5 months after initial diagnosis.

## 3. Discussion

Our case describes pulmonary NC in a middle‐aged male with a never‐smoking history and occupational asbestos exposure, whose therapy was complicated by several hypersensitivity reactions to first line treatments. The case highlights the complexities of diagnosis and therapeutic management of NC. Although the median age at diagnosis of NC is in young adulthood, our case, along with several other cases, has now documented diagnosis across all ages [3]. Due to the limited number of reported cases, no definitive risk factors for the development of primary pulmonary NC have been established to date. Our patient received annual employer‐funded health screening, including chest x‐rays due to possible exposure to asbestos. Although asbestos exposure is linked to many types of lung cancer, its association with NC is uncertain. The patient′s cumulative quantity or duration of exposure is unknown, and whether this history has a contribution to the diagnosis or is incidental cannot be determined.

Owing to the rare incidence, pulmonary NC is often underdiagnosed and lacks standard management. The nonspecific presenting symptoms create a major challenge in determining when it should be included in the differential diagnosis and when to prompt diagnostic IHC staining. In our case, the previously negative pulmonary imaging from 1year prior supports the rapidly progressive nature of NC, further hindering timely and systematic diagnostic approach. Therefore, a low threshold for diagnostic testing in poorly differentiated squamous cell carcinoma is strongly recommended.

Currently, the first‐line diagnostic test for NC is IHC staining for detection of NUT protein. IHC staining with C52 monoclonal antibody has been shown to have high positive and negative predictive values at 100% and 99%, respectively, and with a sensitivity of 87% and specificity of 100% [5]. Positive IHC result meets the WHO diagnostic guideline for NC and should prompt appropriate treatment. Additional testing with FISH or NGS can be used if IHC staining is inconclusive. Both FISH and NGS test for genetic rearrangement, specifically translocation of the *NUTM1* gene. The resulting *BRD4::NUTM1* fusion protein promotes oncogenic proliferation and blocks cellular differentiation, a hallmark of NC [3]. Although the *NUTM1* translocation is the pathognomonic driver of NC, additional genetic alterations may influence tumor biology and therapeutic response. Our patient′s NGS result identified a *CDKN2A* mutation, a tumor suppressor gene whose loss of function has been associated with poorer prognosis and potential resistance to immunotherapy in other cancers [6]. This finding is consistent with a 2025 molecular analysis from the NUT carcinoma registry involving 116 patients, which showed that cell cycle pathway alterations account for 26.0% of cases [7]. It is important to note that homozygous deletion of *CDKN2A* is more commonly associated with squamous cell lung cancers than single‐copy loss [8]. In our case, the zygosity is unknown, and our interpretation of the role of this mutation is speculative.

Given that the initial clinical and histopathologic presentation of pulmonary NC often mimics poorly differentiated squamous cell carcinoma, first‐line therapy frequently follows standard regimens for non‐small cell lung cancer, typically consisting of platinum‐based doublets such as platinum‐paclitaxel or platinum‐etoposide [9]. One might expect immunotherapy to offer a similar benefit. Notably, immune checkpoint inhibitors (ICIs), such as PD‐1/PD‐L1 inhibitors, are now commonly used as a first‐line treatment for advanced squamous cell lung carcinoma [10]. Although few cases have reported transient responses of NC to ICIs, in most cases, treatment response is limited and not sustained [11, 12]. NCs have poor immunogenicity, meaning minimal immune cell infiltration and low PD‐L1 expression [12]. This highlights the distinct molecular features of NC and the need for more specific therapeutic strategies.

As demonstrated in our case, clinical trials of various targeted therapies, particularly BETis, have emerged as promising therapeutic strategies. BETis target the *BRD4::NUTM1* oncogenic fusion protein. Although *BRD4* normally regulates gene expression by binding to acetylated histones, when fused with the *NUTM1* gene, it drives uncontrolled cell proliferation. By binding to BET bromodomains and blocking them from participating in proliferative pathways, BETis regulate oncogenic expression. Earlier studies have shown successful decrease in NUT expression in NC patients after treatment with BETis [13]. However, due to the aggressive disease course, our patient was unable to participate in the clinical trial, a challenge commonly faced by patients with rapidly progressive malignancies. Recent studies have explored other therapeutic options for NC and are summarized in Table 1 [11, 12, 14–20]. However, no treatment has demonstrated a consistent and durable response in advanced NC.

**Table 1:** Overview of targeted and immunotherapeutic approaches under investigation for pulmonary NUT carcinoma.

Therapy	Rationale	Key findings from case reports and clinical trials	Limitations
BET inhibitors	Bet inhibitors competitively block the binding of BRD4 to acetylated histones, suppressing transcription of oncogenes. These inhibitors are also shown to restore cellular differentiation [14]. Synergism with HDAC inhibitors, CDK inhibitors, and p300/CBP inhibitors have been demonstrated in several studies [15–17].	Ye et al. 2025 [14]: A case report of a patient with stage IIIB pulmonary NUT carcinoma showed a complete response achieved within 5 months of NHWD‐870 monotherapy. Lauer et al. 2025 [18]: A Phase Ia/Ib trial of BI894999 showed that 16 out of 42 patients with NUT carcinoma had disease control: one complete response (for 253 days), one confirmed partial response (for 588 days), one unconfirmed partial response, and 13 with stable disease.	Lauer et al. [18] conclude limited clinical effect of BET inhibitors as monotherapy and sought no further evaluation plan of BI894999 as monotherapy for NUT carcinoma.
HDAC inhibitors	Paradoxically, inhibition of histone deacetylase leads to a diffuse increase in histone acetylation, preventing a concentration of acetylation for oncogenic expression, thereby restoring normal cell regulation [15].	Maher et al. 2015 [19]: A case of NUT carcinoma treated with vorinostat and radiation led to a reduction and stabilization of lesions. Treatment was stopped due to thrombocytopenia. CUDC‐907 (NCT02307240) [15]: A small molecule HDAC and phosphoinositide 3‐kinase dual inhibitor is currently in preclinical studies for tumors, including NUT carcinoma.	Data is scarce and limited to preclinical or in vitro studies.
CDK9 inhibitors	CDK9 inhibitors prevent CDK9‐dependent transcription elongation by BRD4‐NUT and promote cell death [20].	Brägelmann et al. 2017 [20]: The in vitro study showed that CDK9 inhibitors disrupt NUT carcinoma cell growth and induce apoptosis.	Evidence is limited to in vitro preclinical data.
P300/CBP inhibitors	These agents inhibit p300/CBP histone acetyltransferase, downregulating expression of oncogenes contained in hyperacetylated megadomains [17].	Zhang et al. 2020 [17]: The in vitro study showed that p300/CBP inhibitor, A‐485, showed specific target activity in NC cell lines and suppressed tumor cell growth. A‐485 also demonstrated cell differentiation along with promotion of cell cycle arrest and induction of apoptosis. Combination with BETi demonstrated synergistic potential.	Evidence is limited to in vitro preclinical data.
PD‐1/PD‐L1 immune checkpoint inhibitors	PD‐1 and PD‐L1 interaction normally turns off programmed cell death and evades immune surveillance. Inhibition of PD‐1/PD‐L1 checkpoint reverses this immune evasion and reactivates T‐cell activity to attack tumor cells [12].	Yang et al. 2025 [11]: A case of pulmonary NUT carcinoma in a 32‐year‐old male treated with sintilimab and chemotherapy showed partial response after two treatment cycles. Of note, subsequent treatment plan also includes BETi targeted therapy and HDACi with chemotherapy. Reintroduction of sintilimab with chemotherapy was discontinued due to neurothlipsis. Chen et al. 2023 [12]: One of two patients who received PD‐L1 inhibitor therapy achieved complete response with atezolizumab and chemotherapy without recurrence or metastasis after 10 months.	NUT carcinoma has poor immunogenicity. Therefore, treatment response is limited and transient. Data from Chen et al. [12] shows mixed outcomes including a patient who had inadequate response to chemotherapy and durvalumab, who later experienced recurrence and extensive metastasis.

*Note:* Combination regimens including two or more of the listed agents utilize complementary immune activities and may demonstrate synergistic effects.

## 4. Conclusion

NC remains a poorly understood malignancy due to its rarity, leading to a lack of data on its epidemiology, risk factors, and treatment. Timely and accurate diagnosis remains challenging due to its nonspecific presentation, resulting in low clinical suspicion, especially in patients outside the typical demographic. Our patient′s complex chemotherapy course and rapid progression underscore the critical need for continued clinical trials to establish effective therapies.

## Funding

No funding was received for this manuscript.

## Conflicts of Interest

The authors declare no conflicts of interest.

## Data Availability

Data sharing is not applicable to this article as no datasets were generated or analyzed during the current study.
